# Mr. Ring, in Answer to Dr. Adams, on Inoculation

**Published:** 1805-10-01

**Authors:** 


### *To the Editors of the Medical and Physical Journal.*

Gentlemen,

In your last Journal, Dr. Adams requests you will "permit an old friend to say a few words in his own defence, when attacked on all sides, and in all manner of ways." He says, "it is not many months since he was severely taken to task, for being candid to Mr. Goldson." It will appear, however, from a reference to your Journal for February last, that the strictures on the conduct of Doctor Adams were occasioned, not by his candour to Mr. Goldson, but by his want of candour to Dr. Mac Donald, and the other friends of vaccination.

Dr. Mac Donald is now in the West Indies, in the service of Great Britain. Had he been in England, it would have been the height of presumption in any man to attempt a defence of his cause. No man is more able to defend himself; or, if his conduct is fairly represented, stands less in need of defence.

Thinking that in his excess of candour to Mr. Goldson, Dr. Adams was deficient in candour to Dr. Mac Donald, I certainly stated my sentiments on the subject; but in a mild way. If it appeared otherwise to Dr. Adams, it must have been from his extreme sensibility on the subject. It is rather surprising, that the severity with which he thought he was treated, did not provoke an answer; but, perhaps, patience and resignation were more advisable, till the first aggression was in some measure forgotten.

Dr. Adams tells us, that when he was so severely taken to task, he "bore it with becoming patience; not thinking it a crime to be civil." To me, however, Dr. Adams appeared to be too civil by half to Mr. Goldson, and very uncivil to Dr. Mac Donald. *Bonis nocet qui parcit malis;* or, to express the same sentiment in other words, *Judex damnatur cum nocens absolvitur.*

But Dr. Adams informs us, he has now been accused by a writer in one of the medical periodical publications, of being uncivil to Mr. Goldson; or, what, he says, is still worse, of mistaking his meaning. Dr. Adams is rather singular in his opinion, that it is a greater crime to mistake a meaning, than to be uncivil.

The two cases of the venereal disease, mistaken for the small-pox, which are brought forward by Dr. Adams, ought to inculcate much caution, before attempts are made to determine the nature of eruptions by inoculation.

Dr. Adams says, happily the instances of small-pox after vaccination, or inoculation, are rare. It is, therefore, a little remarkable, that just afterwards, speaking of the occurrence of the small-pox a second time, he tells us, such instances are now become so numerous, and *well authenticated,* that he was surprised to find, in your former Number, the case of Miss Price, which he had before related, brought as a fresh communication.

The fact is, that the Rev. Mr. Jenner had previously promised to inform me of any cases of the small-pox occurring a second time; and when this case occurred, he communicated it to me for publication, as well as to Dr. Adams. "The little addition at the close, in which Mr. Travers's firebrands are introduced," came from the pen of Mr. Jenner; but I saw no reason to omit it, merely because the same expression had been introduced once before.

Bis repetita placet, decies repetita placebit.

No error, into which Dr. Adams has fallen in his present letter can surprise those who have read his former publications on this subject, particularly his little tract, which the Editors of the Medical and Chirurgical Review consider as an *ad captandum.* He there affirms, that *no objections have been made against vaccination but in England?* He affirms that the practice is spread thro' *Africa;* but he has not informed us on what authority this assertion rests.

He represents England as the only nation that speaks evil of this blessing; while other countries are availing themselves of it, and laughing at her folly. This is a proof, that Dr. Adams's information is not very correct. He undertakes to answer for the goodness of Mr. Goldson's intention; but it is always sufficient for any man, to answer for the goodness of his own.

He

He also admits Mr. Travers's good intention, and the purity of the motives by which he was actuated, when he pronounced his eloquent Philippic against variolous inoculation at the Crown and Anchor. In that Philippic, if he did not convert some people, he spoke daggers to their souls. He tells us, Boerhaave enjoins every Physician to read Sydenham's Treatise on the small-pox, thirteen times over; and observes, that by so doing, we find "inoculation was not necessary to spread the *disease universally* through London, under *certain constitutions of the atmosphere.*"

He says, Mr. Travers could not be aware, "that the small-pox is so *constantly epidemic* in London, that no person who has not had the disease thinks himself safe in passing a night in the metropolis." Here, however, is a clear distinction between the statement of Sydenham and that of Dr. Adams; and it is not necessary to read the two authors *thirteen times*, in order to discover it. In Sydenham's time, there were particular years in which the small-pox was epidemic; and it was supposed, that a certain constitution of the atmosphere was necessary to produce that universal scourge. Now, a resolution of the Governors of the Pest House at Pancras, or even the active exertions of a single individual, are at any time sufficient to produce it.

Hoc fonte derivata labes,
In patriam populumque fluxit.

Dr. Adams endeavours to win Mr. Travers over to his cause, by appealing to that love of liberty which reigns in his breast. He boasts of having been *acquainted* with Mr. Travers thirty years. It is, however, evident that he does not *know* him yet; otherwise he would know, that his love of liberty is tempered with a love of justice; and that he never takes the liberty of doing to others, what he would not have them do to him. He is even so unfashionable as to think, that instead of reading Sydenham thirteen times, every physician ought to read Sydenham twelve times, and his bible once. He may there learn that salutary lesson, *Thou shalt do no murder.*

Dr. Adams is of opinion, that Mr. Travers is excuseable for not knowing how prevalent the small-pox is in London, because he is not a medical man, and because, forsooth, he has not read Sydenham. He has, however, read the great volume of Nature, which Sydenham read before him, and which is widely opened in this metropolis every day. He there sees the baneful effects of avarice, ambition, and a love of fame. He there sees a practice tolerated, which, it is agreed on all hands, ever has been injurious to the public; and which, since the discovery of vaccination, ever will.

Dr. Adams says, he knows Mr. Travers well enough to be *satisfied of his love of liberty,* and *of the genuine sentiments of affection with which his heart is stored.* He therefore supposes, that if he saw a child whose parents would not consent to vaccination, he would rather inoculate him for the small-pox, than suffer him to be exposed to the horrors of the natural disease.

Happily for Mr. Travers, he is not a medical man, and therefore not reduced to such a dilemma, as to be obliged to determine this point. It must, however, be recollected, that every one who recommends inoculation of the small-pox, even under such circumstances, countenances the practice, and diffuses the natural infection. Dr. Adams, indeed, tells us, that London is never free from the small-pox; and it is never likely to be free, if prejudice, obstinacy, and self-interest are suffered to continue variolous inoculation without restraint.

Even Dr. Adams, however, admits, that since the invaluable discovery of Dr. Jenner is made, there can be no objection to certain legal restraints on the inoculation of the small-pox. But he attributes the late prevalence of the disease too much to the *constitution of the air,* and too little to the *constitution of the Small-pox Hospital.*

I know not any apology that has been offered for continuing variolous inoculation in London, which may not be offered for continuing it in Bath; yet the apology for continuing the practice there has been treated by Dr. Parry with the contempt it deserves. In the Bath Journal for August 19, speaking of those who still persisted in it, he says, "Let these persons go to their pillows with this reflection, and sleep on them with what comfort they may; and let them, by publicly boasting of the success of their own efforts to spread this scourge of mankind, in spite of all the efforts of the friends of humanity to expel it for ever from the face of the earth, afford a further proof, if any were still wanting, that insolence usually accompanies vice."

With respect to the Small-pox Hospital, if variolous inoculation is to be permitted at all, let it be confined to the walls of that habitation; and if there be any advocate for the practice, let him boast of the success of his efforts there also: *Illâ se jactet in aulâ.*

As

As to vaccination, it is a practice which requires not a retired and solitary situation, but one that is central and populous, in order to accommodate the public. But the most expeditious, and the most effectual method of promoting the practice, and thereby hastening the extermination of the small-pox, is to visit and convert the poor, and to vaccinate from house to house. Much experience has proved, that there are numbers of people so indifferent to the welfare of their children, as not to take the trouble of going any distance on that account, who, nevertheless, will consent to have them inoculated at their own homes.

I lately received a letter from a physician in the country, in which he says, "The pest-house of St. Pancras *must* be put under some restrictions, or vaccination may go again to its native meadows. Even at this distance from the metropolis, I am doomed to see the horrid effects of promiscuous inoculation. Two young persons brought the small-pox hither from London; one of them escaped with life, but the other, a fine young fellow, servant to the Archbishop of Canterbury, is just deposited in our church-yard.

"The disease is also beginning to spread in the neighbouring villages; which keeps my lancet fully employed. London must be considered as the focus of antivaccinism. The mischief arising from a single individual sent out of the Small-pox Hospital, and let loose on society, exceeds all calculation. What was the lance of an Ajax, compared to that of a Wachsel?"

One of the first measures pursued in India, after the introduction of the cow-pock, was to prohibit variolous inoculation. The same policy appears requisite here; unless our population is too great. Indeed, it is peculiarly necessary in this overgrown metropolis, on account of the greater difficulty of discovering and avoiding the infected subjects. Besides, there are multitudes of the inhabitants of London, who are less under the influence of their neighbours, than in country towns and villages. If, therefore, such persons are resolved to continue obstinate; if they are deaf to the call of humanity, and will not listen to the voice of reason, let them retire to hospitals and lazarettoes, when the pest is in their families, and let bounds be assigned to them, which they shall not pass. This is what the safety of the people requires, and what the public have a right to demand.

Dr. Adams mentions other measures, and seems to lay hold of every twig, and every straw, in order to support the inoculation of the small-pox; and, consequently, the hospital destined for that purpose. He says, early vaccination is the only security for an inhabitant of London. But even vaccination, if attempted at the moment of birth, is not an absolute security. The operation may fail the first time, and perhaps the second time; and, to use the pathetic language of Dr. Adams, the "lovely little infant," the "innocent infant," may be "coolly condemned to all the horrors of that dreadful disease, the small-pox."

If we cannot have an absolute security, let us at least have a comparative security; let us have such a security as we can obtain. It is fresh in the memory of every one, that while vaccination increased, the small-pox decreased. Were the inoculation of the small-pox interdicted, or subject to proper legal restraints, time and reflection would assist the friends of vaccination in reconciling the public mind to the practice; and, like Fabius, we should conquer by delay.

Dr. Adams subjoins some remarks on the eruptions that have lately been common. I believe they have, in former times, been equally common, but not equally submitted to the inspection of medical men. He justly observes, that at another time, the venereal cases which he describes, would scarcely have found their way to the Small-pox Hospital. There is another eruptive disease, on which I long ago published some observations. The subject was introduced by Mr. Badger; who spoke of an infectious disease of the scalp, which was considered as a non-descript. Dr. Willan afterwards remarked to me, that he saw no reason for calling those eruptions by any other than their usual names, tinea, or favus; and in this opinion I readily acquiesce.

One case of this species of eruption, which began in the face, and spread over the neck and chest, occurred in a child in Midford Place, Tottenham Court Road, and was pronounced to be the small-pox by Mr. Walker, of St. James's Street; and Mr. Birch, with whom he called on the child the next day, confirmed that opinion. The case was therefore reported to me, as a case of the small-pox after the cow-pox. Having inquired into the particulars, I was surprised to hear the opinion given. Two or three small eruptions first appeared about the temples, and by the next day the eye-lids were so much swollen, that the child was blind. The mother then applied a blister to the nape of the neck, which soon reduced the swelling of the eye-lids, and occasioned a considerable eruption round the part where it was applied. It likewise reached, without interruption, almost to the navel; but there was not the least trace of it below the navel; nor on any of the extremities.

It is worthy of notice, that the two antivaccinists, who, in the present instance, spread an alarm, notwithstanding they assured the child's mother, that the case was the small-pox, and terrified all the mothers in the neighbourhood, whose children had been vaccinated, did not, as usual, convene their medical friends, nor take matter. Hence it appears, that whatever alarm they wished to excite, they did not wish the affair should undergo any farther investigation.

Having seen the case, I was surprised at the opinion given; and the next day requested Dr. Willan and Dr. Adams to see it with me, to prevent misrepresentation. Dr. Willan declared it to be that species of eruption called *favus*. It is extremely common; and is generally occasioned by cold. In the present instance it was very well accounted for by a Lodger; who informed me, that the child's mother had placed him on the threshold, in a very cold and windy day; and left him there the whole time, while she scoured the passage.

The day after Dr. Willan and Dr. Adams had visited him with me, his mother, again carried the child to Mr. Walker; who positively assured her that the case was the small-pox; and gave her physic for him, on that account. Soon afterwards, however, a fresh eruption of a similar kind took place, on a fresh exposure to cold, and the mother carried him to Mr. Wachsel; who told her the small-pox never continued coming out for a month. This remark, and his unquestionable judgement in such cases, at length convinced her of the error into which she had been led.

Dr. Adams has added one case to those already on record, of the small-pox mitigated by vaccination; and four in one family, of local pustules; three of which were attended with fever. This alone, if we had no other evidence of the kind, is sufficient to refute the vulgar error, that those who have had the small-pox, although they may again have pustules, from contact of matter, can never have any constitutional indisposition from this cause.

[ To be continued. ]

